# Hospitalizações e Mortalidade Hospitalar por Insuficiência Cardíaca no Brasil: Um Panorama Atualizado

**DOI:** 10.36660/abc.20250284

**Published:** 2025-06-18

**Authors:** Adriana Lopes Latado

**Affiliations:** 1 Universidade Federal da Bahia Faculdade de Medicina da Bahia Salvador BA Brasil Universidade Federal da Bahia - Faculdade de Medicina da Bahia, Salvador, BA – Brasil; 2 Universidade Federal da Bahia Hospital Universitário Professor Edgard Santos Salvador BA Brasil Universidade Federal da Bahia - Hospital Universitário Professor Edgard Santos, Salvador, BA – Brasil

**Keywords:** Insuficiência Cardíaca, Hospitalização, Prognóstico, Mortalidade Hospitalar

A insuficiência cardíaca (IC) representa uma carga crescente de doença e custos de saúde, tanto globalmente quanto no Brasil; é a via final comum de várias doenças cardiovasculares, incluindo condições específicas e bem definidas, como a doença isquêmica do coração. Dados de prevalência e incidência mostram aumentos progressivos ao longo das últimas décadas, com a prevalência atual de IC estimada em 2,5% entre adultos nos Estados Unidos, e projetada para atingir 3,0% até 2030.^1,2^ As internações hospitalares relacionadas à IC também estão aumentando nos Estados Unidos. Enquanto isso, a mortalidade hospitalar ajustada diminuiu, de 6,8% em 2002 para 4,9% em 2016, um padrão consistente entre faixas etárias, sexo e categorias raciais/étnicas.^1^

No Brasil, a prevalência autorreferida de IC é estimada em 1,1% entre adultos acima de 18 anos e 3,3% em indivíduos acima de 60 anos.^2^ Em relação às hospitalizações e à mortalidade hospitalar, estudos de tendência temporal indicam uma redução no número de internações relacionadas à IC, com a mortalidade hospitalar variando de 9% a 17%, também apresentando uma tendência de queda, especialmente na última década.^1,3,4^ No entanto, os dados nacionais ainda são escassos, com limitações na representatividade populacional e necessidade de atualizações regulares.

O artigo de Girardi et al.,^5^ publicado nesta edição, examina as tendências temporais das internações relacionadas à IC em adultos com idade ≥40 anos no Brasil entre 2000 e 2021, utilizando dados do DATASUS e estratificando por idade e sexo. O estudo também analisa as tendências de mortalidade hospitalar ao longo desse período de 22 anos. Os autores relatam uma queda constante nas internações por IC tanto entre homens quanto mulheres em todas as faixas etárias. A redução percentual média anual nas taxas de internação entre os homens variou de 6,7% (40–49 anos) a 8,1% (≥80 anos). Entre as mulheres, a queda variou de 7,5% (70–79 anos) a 8,3% (50–59 anos). Em contraste, as taxas de mortalidade hospitalar apresentaram uma tendência de aumento em todas as faixas etárias e em ambos os sexos de 2000 a 2021. Entre os homens, o aumento percentual médio anual variou de 1,8% (40–49 anos) a 3,6% (≥80 anos). Entre as mulheres, o crescimento variou de 3,1% (≥80 anos) a 3,5% (60–79 anos). Algumas variações adicionais foram observadas em análises específicas por subperíodo e idade, mas sem impacto significativo nos achados gerais.^5^

Os achados relatados no artigo^5^ refletem o cenário epidemiológico global da IC? Dados dos Estados Unidos mostram que as internações hospitalares relacionadas à IC aumentaram de 1,06 milhão em 2008 para 1,27 milhão em 2018, com taxas mais altas entre grupos raciais e étnicos sub-representados em estudos clínicos, como as populações negra e hispânica. As internações aumentaram em todos os subtipos de IC com base na fração de ejeção do ventrículo esquerdo (FEVE), sendo que homens foram mais frequentemente internados por IC com fração de ejeção reduzida (ICFEr) e mulheres por IC com fração de ejeção preservada (ICFEp). Entre 2002 e 2016, a mortalidade hospitalar diminuiu de 6,8% para 4,9%, uma tendência consistente entre faixa etária, sexo e raça.^1^

Os dados sobre IC na Europa variam significativamente entre os países, dependendo dos níveis de renda, do acesso à saúde e dos fenótipos de IC com base na FEVE. No Reino Unido, um estudo retrospectivo populacional encontrou um aumento nas internações hospitalares por IC entre mulheres de 1998 a 2017, enquanto as taxas permaneceram estáveis entre os homens. Indivíduos de origens socioeconômicas mais baixas apresentaram uma tendência de aumento nas internações, um padrão não observado entre as populações mais desfavorecidas.^6^ Um estudo italiano relatou uma redução nas hospitalizações por IC ao longo de um período de 38 anos (1977–2014) entre indivíduos com mais de 65 anos,^7^ enquanto na Suécia, uma análise baseada na idade encontrou um aumento nas internações por IC em adultos mais jovens, mas não em grupos etários mais velhos (55–84 anos). Além disso, a mortalidade hospitalar diminuiu em todas as faixas etárias de 1987 a 2001, mas estabilizou-se posteriormente.^8^

Fica evidente que inconsistências na epidemiologia e no prognóstico da IC podem existir entre países e até mesmo dentro de regiões de grandes nações, independentemente da etiologia ou dos fenótipos da síndrome. Essas diferenças são multifatoriais.^9^ No entanto, está bem estabelecido que as terapias modificadoras da doença para IC – especialmente para ICFEr – impactaram significativamente a mortalidade geral e reduziram as hospitalizações nas últimas três décadas.^10,11^ Se esse for o caso, as hospitalizações e reinternações atuais provavelmente estão ocorrendo em pacientes clinicamente mais graves, nos quais desfechos adversos, como morte hospitalar, são mais frequentes.

A falta de uma melhoria uniforme no prognóstico da IC é complexa e vai além da eficácia da terapia farmacológica orientada por diretrizes. Fatores como acesso à saúde, disponibilidade de medicamentos, renda regional ou nacional e o status socioeconômico e educacional da população influenciam significativamente os desfechos reais de estratégias que se mostraram eficazes em ensaios clínicos randomizados.

O estudo^5^ traz uma contribuição valiosa e oportuna para a compreensão do prognóstico da IC no Brasil. Embora os dados epidemiológicos baseados no DATASUS sejam limitados à população atendida pelo Sistema Único de Saúde (SUS), é importante destacar que aproximadamente 84% dos brasileiros dependem exclusivamente do SUS – um percentual ainda maior nas regiões norte e nordeste.^12^ Estudos ecológicos sobre IC são urgentemente necessários para expandir o conhecimento sobre sua epidemiologia e seu prognóstico. Esses estudos devem abordar as diversas realidades dessa síndrome complexa e fornecer dados que reflitam a diversidade regional, étnica, cultural e socioeconômica do Brasil.
