# Supplementary material for: Hospitalizações e Mortalidade Hospitalar por Insuficiência Cardíaca no Brasil: Um Panorama Atualizado
Source: Arq Bras Cardiol. 2025 Jun 18;122(6):e20250284. [Article in Portuguese] doi: 10.36660/abc.20250284 (PMC12212390; doi:10.36660/abc.20250284)
Supplement: Translation

# Translation: Hospitalizations and In-Hospital Mortality from Heart Failure in Brazil: An Updated Overview

**DOI:** 10.36660/abc.20250284i

**Authors:** Adriana Lopes Latado

**Affiliations:** 1 Salvador BA Brazil Universidade Federal da Bahia - Faculdade de Medicina da Bahia, Salvador, BA – Brazil; 2 Salvador BA Brazil Universidade Federal da Bahia - Hospital Universitário Professor Edgard Santos, Salvador, BA – Brazil

**Keywords:** Heart Failure, Hospitalization, Prognosis, Hospital Mortality

Heart failure (HF) accounts for a growing burden of disease and healthcare costs both globally and in Brazil. It represents the final common pathway of several cardiovascular diseases, including specific and well-defined conditions such as ischemic heart disease. Prevalence and incidence data have shown progressive increases over recent decades, with the current HF prevalence estimated at 2.5% among U.S. adults, and projected to reach 3.0% by 2030.^1,2^ HF-related hospital admissions are also on the rise in the United States. Meanwhile, adjusted in-hospital mortality has declined, from 6.8% in 2002 to 4.9% in 2016, consistent across age groups, sex, and racial/ethnic categories.^1^

In Brazil, the self-reported prevalence of HF is estimated at 1.1% among adults over 18 years old and 3.3% in individuals over 60.^2^ Regarding hospitalizations and in-hospital mortality, temporal trend studies indicate a decline in the number of HF-related admissions, with hospital mortality ranging from 9% to 17%, also showing a decreasing trend, especially over the past decade.^1,3,4^ However, national data remain scarce, with limitations in population representativeness and a need for regular updates.

The article by Girardi et al.,^5^ published in this issue, examines the temporal trends of HF-related hospitalizations in adults aged ≥40 years in Brazil between 2000 and 2021, using data from DATASUS and stratifying by age and sex. It also analyzes in-hospital mortality trends over this 22-year period. The authors report a steady decline in hospitalizations for HF among both men and women across all age groups. The average annual percentage decrease in hospitalization rates among men ranged from 6.7% (ages 40–49) to 8.1% (ages ≥80). Among women, the decline ranged from 7.5% (ages 70–79) to 8.3% (ages 50–59). In contrast, in-hospital mortality rates showed an upward trend across all age groups and in both sexes from 2000 to 2021. Among men, the average annual increase ranged from 1.8% (ages 40–49) to 3.6% (ages ≥80). Among women, the increase ranged from 3.1% (ages ≥80) to 3.5% (ages 60–79). Some additional variations were observed in sub-period and age-specific analyses, but without major impact on the overall findings.^5^

Do the findings reported in article^5^ reflect the global epidemiological landscape of HF? U.S. data show that HF-related hospitalizations increased from 1.06 million in 2008 to 1.27 million in 2018, with higher rates among racially and ethnically underrepresented groups in clinical studies, such as Black and Hispanic populations. Hospitalizations increased across all HF subtypes based on left ventricular ejection fraction (LVEF), with men more frequently admitted for HF with reduced ejection fraction (HFrEF), and women for HF with preserved ejection fraction (HFpEF). Between 2002 and 2016, in-hospital mortality declined from 6.8% to 4.9%, a trend consistent across age, sex, and race.^1^

In Europe, data vary significantly between countries depending on income levels, access to healthcare, and HF phenotypes based on LVEF. In the United Kingdom, a population-based retrospective study found increasing HF hospitalizations among women from 1998 to 2017, while rates remained stable in men. Individuals from lower socioeconomic backgrounds showed a trend toward increased admissions, a pattern not observed among the most disadvantaged populations.^6^ An Italian study reported a decline in HF hospitalizations over a 38-year period (1977–2014) among individuals over 65,^7^ whereas in Sweden, an age-based analysis found increased HF admissions in younger adults, but not in older age groups (55–84 years). Furthermore, hospital mortality declined across all age groups from 1987 to 2001, but plateaued thereafter.^8^

It is clear that inconsistencies in HF epidemiology and prognosis can exist across countries and even within regions of large nations, regardless of the syndrome's etiology or phenotypes. These differences are multifactorial.^9^ However, it is well established that disease-modifying therapies for HF – especially HFrEF - have significantly impacted overall mortality and reduced hospitalizations over the past three decades.^10,11^ If this is the case, current hospitalizations and readmissions are likely occurring in clinically more severe patients, in whom adverse outcomes, such as in-hospital death, are more frequent.

The lack of a uniform improvement in HF prognosis is complex and extends beyond the efficacy of guideline-directed pharmacologic therapy. Factors such as healthcare access, medication availability, regional or national income, and the population's socio-economic and educational status significantly influence the real-world outcomes of strategies that have proven effective in randomized clinical trials.

The study^5^ makes a valuable and timely contribution to understanding HF prognosis in Brazil. While DATASUS-based epidemiological data are limited to the population served by the Brazilian Unified Health System (SUS), it is important to note that approximately 84% of Brazilians currently rely exclusively on SUS – a figure even higher in the north and northeast regions.^12^ More ecological studies on HF are urgently needed to expand knowledge about its epidemiology and prognosis. These studies should address the diverse realities of this complex syndrome and provide data reflective of Brazil's regional, ethnic, cultural, and socioeconomic diversity.
